# Safety Evaluation of *Scutellaria baicalensis*: Acute and 26-Week Repeated-Dose Oral Toxicity Studies in Rats

**DOI:** 10.3390/toxics14100919

**Published:** 2026-10-17

**Authors:** Jungbin Song, Hocheol Kim

**Affiliations:** Department of Herbal Pharmacology, College of Korean Medicine, Kyung Hee University, 26, Kyungheedae-ro, Dongdaemun-gu, Seoul 02447, Republic of Korea; jbsong@khu.ac.kr

**Keywords:** *Scutellaria baicalensis*, Scutellariae Radix, toxicity, safety, minimum lethal dose, no-observed-adverse-effect level, no-observed-effect level

## Abstract

*Scutellaria baicalensis* root is widely used in traditional medicine and functional foods, but information on its long-term toxicity remains limited. This study evaluated the acute and 26-week repeated-dose oral toxicity of *S. baicalensis* root extract (SBE) in male and female Sprague–Dawley rats under Good Laboratory Practice conditions. In the acute study, SBE was administered once at 2500 or 5000 mg/kg, followed by 14 days of observation. In the repeated-dose study, SBE was administered at 500, 1000, or 2000 mg/kg/day for 26 weeks, followed by a 4-week recovery period. No mortality occurred in either study. Acute administration caused only transient, non-adverse clinical signs, and the minimum lethal dose was >5000 mg/kg. Repeated administration at 2000 mg/kg/day increased relative liver weight and urinary ketone grades in both sexes, while absolute liver weight and serum triglycerides increased in females. These findings were considered treatment-related but non-adverse, without corroborating clinical, serum biochemical, or histopathological evidence of organ injury. The no-observed-effect level and no-observed-adverse-effect level were 1000 and 2000 mg/kg/day, respectively, and no target organ of toxicity was identified. These findings provide preclinical safety information for acute and long-term oral exposure to SBE.

## 1. Introduction

*Scutellaria baicalensis* Georgi, a perennial plant of the Lamiaceae family commonly known as Baikal skullcap or Chinese skullcap, is widely distributed in East Asia. Its dried root, Scutellariae Radix, has been used for centuries in traditional East Asian medicine for the treatment of dysentery, hypertension, and various inflammatory and infectious diseases [1]. It is also widely used worldwide in herbal medicines and functional food prod-ucts [2,3,4]. Scutellariae Radix is rich in flavonoids, particularly baicalin, wogonoside, bai-calein, and wogonin [4]. These constituents have been extensively investigated for their anti-inflammatory, antioxidant, neuroprotective, hepatoprotective, immunomodulatory, and anticancer activities [3,4,5,6,7].

Despite its long history of use, the toxicological profile of Scutellariae Radix has not been fully established. Acute oral toxicity studies of aqueous, hydroalcoholic, and fermented extracts of Scutellariae Radix reported no mortality or treatment-related toxicity at the tested doses [8,9,10]. However, these studies provide limited information on the effects of prolonged exposure.

Potential safety concerns have also emerged from clinical observations. Sporadic cases of clinically apparent liver injury have been reported following the use of *S. bai-calensis*-containing products [11]. A recent literature review identified 16 suspected cases of *S. baicalensis*-associated drug-induced liver injury; however, most involved multi-ingredient preparations, making the specific contribution of *S. baicalensis* difficult to determine [12].

Preclinical studies have further raised concerns regarding potential hepatic, renal, and cardiac effects. A 26-week repeated-dose study of an ethanol extract of *S. baicalensis* reported inflammatory changes in the liver at 2500 mg/kg/day, which resolved after treatment withdrawal [13]. High-dose exposure to major flavonoids of Scutellariae Radix has also been associated with organ-specific toxicity, including renal injury and fibrosis with baicalin and cardiac injury with wogonin [14,15]. More recently, repeated administration of baicalin at 4000 mg/kg/day for 28 days produced mild hepatic and renal alterations in mice [16]. In addition, a 13-week repeated-dose study of a combined extract of *S. baicalensis* and *Pueraria lobata* showed centrilobular hepatocellular hypertrophy and increased relative liver weight at high doses, although these changes were considered adaptive and reversible [17].

These findings suggest that repeated or high-dose exposure to *S. baicalensis* and its constituents may affect the liver, kidney, and heart. However, comprehensive long-term toxicity data for *S. baicalensis* root extract remain limited, and key toxicological parameters, including dose–response relationships, no-observed-effect levels (NOELs), no-observed-adverse-effect levels (NOAELs), potential target organs, and the reversibility of treatment-related effects, have not been adequately defined under regulatory-compliant conditions.

To address these gaps, the present study evaluated the acute and 26-week repeated-dose oral toxicity of *S. baicalensis* root extract in male and female Sprague–Dawley rats, including a 4-week recovery period. The studies were conducted under Good Laboratory Practice (GLP) conditions in accordance with the toxicity testing guidelines of the Korean Ministry of Food and Drug Safety (MFDS) to assess general toxicity, determine NOEL and NOAEL values, identify potential target organs, and evaluate the reversibility of treatment-related effects.

## 2. Materials and Methods

### 2.1. Preparation of S. baicalensis Extract (SBE)

Dried roots of *S. baicalensis* from Shandong province, China, were purchased from Dong Kyung Pharm Co., Ltd. (Seoul, Republic of Korea). The roots were extracted with 70% ethanol for 3 h at 80 °C under reflux conditions, and the resulting extract was filtered. The filtrate was then concentrated under reduced pressure using a rotary evaporator at 45 °C and lyophilized with a freeze dryer to yield a yellow powder. The extraction yield was 26.08%. A single batch of SBE was used in both the single-dose and 26-week repeated-dose toxicity studies. The contents of the major flavonoids in this batch, as determined using a validated high-performance liquid chromatography method, were 19.8% baicalin, 3.78% baicalein, 3.82% wogonoside, and 0.62% wogonin (all *w*/*w*, on a dry extract basis).

### 2.2. Animals

Seven-week-old and five-week-old Sprague–Dawley (SD) rats were obtained from Koatech (Pyeongtaek, Gyeonggi-do, Republic of Korea) for the acute and the 26-week repeated-dose oral toxicity studies, respectively. Toxicity tests were conducted at Chemon Inc. (Yongin, Gyeonggi-do, Republic of Korea) and complied with the Korea Food and Drug Administration (KFDA; now the Korean MFDS)’s Standard for Toxicity Study of Pharmaceuticals (No. 2005-60) and GLP Regulation for Non-clinical Laboratory Studies (No. 2005-79). The acute and the 26-week repeated-dose oral toxicity studies were approved by the Institutional Animal Care and Use Committee of the Preclinical Research Center, Chemon Inc. (Approval Nos.: 07-RA-136, 07-RR-137). Animals were allowed free access to standard food and water and were acclimatized to facility conditions for 7 days prior to the experiments. The animals were kept under the following conditions: 23 ± 3 °C, 55 ± 15% relative humidity, and a 12-h light-dark cycle (08:00 lights on–20:00 lights off).

### 2.3. Acute Oral Toxicity Study

Fifteen female and fifteen male SD rats were ranked by body weight and allocated to three groups per sex using a serpentine allocation method: control, SBE 2500 mg/kg, and SBE 5000 mg/kg groups, with five rats in each group. The rats were fasted overnight before dosing. SBE was dissolved in sterile water for injection (vehicle) and administered orally at a volume of 15 mL/kg directly into the stomach via an oral gavage needle in a single dose. The control group was administered the vehicle following the same procedure. The rats were continuously monitored for 1 h post-administration, and then hourly for up to 6 h for any signs of mortality or clinical symptoms. Observations continued at least once daily for 14 days post-administration. Body weights of all rats were measured just before administration, and on days 1, 3, 7, and 14 post-administration. On day 14 post-administration, the rats were anesthetized with ether inhalation and euthanized by exsanguination through the posterior vena cava and abdominal aorta. A gross necropsy was performed to examine the external surfaces and organs.

### 2.4. Twenty-Six-Week Repeated-Dose Oral Toxicity Study

#### 2.4.1. Group Allocation and Dosing

Fifty female and fifty male SD rats with body weights close to the mean were ranked by body weight. The rats were then allocated to four main groups (10 rats per group) and two recovery groups (5 rats per group) for each sex to balance initial body weights across groups. The sample size was determined in accordance with the toxicity testing guidelines (Notification No. 2005-60) of the KFDA, now the Korean MFDS. All rats underwent 26 weeks of treatment with once daily dosing, with those in the recovery groups undergoing an additional 4 weeks of observation without further dosing. One main group and one recovery group were designated as controls and received the vehicle (sterile water for injection) at a dosing volume of 10 mL/kg via oral gavage needle. The three other main groups received SBE at doses of 500, 1000, and 2000 mg/kg/day, respectively, and the additional recovery group received SBE at 2000 mg/kg/day. All treatments were administered under the same conditions (volume and route) as the control group.

Dose levels were selected based on the results of our acute oral toxicity study and a 2-week preliminary repeated-dose study. No mortality was observed following a single oral dose of 5000 mg/kg. In the preliminary repeated-dose study, administration at 1000 and 2000 mg/kg/day produced no mortality or clinical signs and had no effect on body-weight gain. Based on these preliminary findings, 2000 mg/kg/day was selected as the high dose, corresponding to 20 times the pharmacologically effective dose of 100 mg/kg identified in our previous pharmacological studies in rats (unpublished data). The middle and low doses of 1000 and 500 mg/kg/day, respectively, were selected using twofold descending intervals.

#### 2.4.2. Clinical Signs

During the administration and recovery periods, mortality, the types of clinical signs observed, the onset days of these signs, and their severity were monitored daily. Each observation was recorded individually for every rat.

#### 2.4.3. Body Weight

The body weight of all rats was measured on the day dosing began, and subsequently once a week, as well as on the day before and on the day of necropsy. The body weight at necropsy was measured after overnight fasting.

#### 2.4.4. Food and Water Consumption

Food consumption was measured on the day dosing began, weekly until week 13 post-dosing, once every four weeks from week 14 to 26, and on the day before necropsy. For the recovery group after week 26, measurements were taken bi-weekly. A predetermined amount of food was provided per cage, and the remaining quantity was measured the following day. The difference was calculated to determine the average intake per rat per day (g/rat/day).

Water consumption was measured at the same frequency as food consumption. A predetermined amount of water was provided per cage, and the remaining quantity was measured the following day. The difference was calculated to determine the average intake per rat per day (g/rat/day).

#### 2.4.5. Ophthalmologic Examination

Upon group allocation, the appearance of the eyes of all rats was observed with the naked eye. In the final week of observation, for rats in both the control and high-dose groups (2000 mg/kg/day), the appearance of the eyes was again examined with the naked eye. Mydriasis was induced in both eyes using homatropine hydrobromide (Samil Pharm, Seoul, Republic of Korea) administered as eye drops. Then, observations and photography of the anterior segment, intermediate transparent media, and fundus were conducted using a fundus camera (Genesis, Kowa Co., Ltd., Nagoya, Japan). Since no abnormalities were observed in the eyes of the high-dose group, fundus examinations were not extended to the other lower dose groups.

#### 2.4.6. Urinalysis

Urinalysis was performed during the last week of the 26-week administration period in the main groups and during the last week of the 4-week recovery period in the recovery groups. In the main groups, the five rats with the lowest identification numbers within each group and sex were selected for urinalysis. In the recovery groups, all five rats of each sex per group underwent urinalysis. The rats from each group were individually housed in metabolic cages for urine collection. Fresh urine collected over a period of 3–4 h was applied to a test strip (Multistix 10SG, Bayer, Elkhart, IN, USA) and analyzed with an automated urine analyzer (CliniTek 100, Bayer) to measure the following parameters: glucose, bilirubin, ketone bodies, specific gravity, occult blood, pH, protein, urobilinogen, nitrite, and leukocytes. The fresh urine was also visually examined for color, then centrifuged at 1500 rpm for 5 min to prepare for urine sediment examination. The sediment was stained using the Sternheimer–Malbin method and observed under a microscope for red blood cells, white blood cells (WBCs), epithelial cells, and casts. Additionally, the total urine volume collected over 24 h was measured.

#### 2.4.7. Necropsy and Organ Weight

On the day of necropsy, fasting rats were anesthetized via ether inhalation. Blood was collected from the posterior vena cava for hematological and biochemical analyses. Then, the abdominal aorta and posterior vena cava were severed to exsanguinate and euthanize the animals. A gross necropsy was performed to examine the external surfaces, subcutaneous areas, head, thoracic and abdominal cavities, and all organs therein. Organ weights were measured, and tissues were fixed for further analysis.

#### 2.4.8. Hematology

The collected blood was placed into a CBC bottle containing EDTA-2K, and the following parameters were measured using an automated blood analyzer (ADVIA 2120, Siemens, Tarrytown, NY, USA): WBC count, red blood cell count, hemoglobin concentration, hematocrit, mean corpuscular volume, mean corpuscular hemoglobin, mean corpuscular hemoglobin concentration, red cell distribution width, hemoglobin concentration distribution width, platelet count, mean platelet volume, and reticulocyte count. A differential WBC count was conducted for neutrophils, lymphocytes, monocytes, eosinophils, basophils, and large unstained cells.

#### 2.4.9. Serum Biochemistry

Blood was placed into a vacutainer tube containing a clot activator (Insepack, Sekisui, Tokyo, Japan) and allowed to clot at room temperature for 15–20 min before being centrifuged at 3000 rpm for 10 min to obtain serum. The serum was analyzed using an automated clinical chemistry analyzer (AU400, Olympus, Tokyo, Japan) for the following parameters: aspartate aminotransferase, alanine aminotransferase, alkaline phosphatase, blood urea nitrogen, creatinine, glucose, total cholesterol, total protein, creatine phosphokinase, albumin, total bilirubin, triglycerides, inorganic phosphorus, calcium ion, and albumin/globulin ratio. Electrolyte levels, including sodium, potassium, and chloride ions, were measured using an electrolyte analyzer (Ciba-Corning 644 Na/K/Cl Analyzer, Ciba-Corning, Medfield, MA, USA).

#### 2.4.10. Plasma Coagulation

Blood was placed into a microtube containing 0.2 mL of 3.2% sodium citrate and then centrifuged at 3000 rpm for 10 min to obtain plasma. The coagulation times, specifically the prothrombin time and activated partial thromboplastin time (aPTT), were measured in seconds using a coagulation analyzer (ACL 100, Instrumentation Laboratory, Bedford, MA, USA) through nephelometric analysis.

#### 2.4.11. Histopathology

The organs of all rats were fixed in 10% neutral buffered formalin solution, except for the eyes, which were fixed in Davidson’s solution, and the testes and epididymides, which were fixed in Bouin’s solution. The following organs were included: testes, epididymides, seminal vesicles, prostate, ovaries, uterus, vagina, urinary bladder, spleen, stomach, pancreas, duodenum, jejunum, ileum, cecum, colon, rectum, mesenteric lymph nodes, adrenal glands, kidneys, liver, skeletal muscle (femoral muscle), sciatic nerve, femur, mandibular lymph nodes, salivary glands, sternum, thymus, heart, lungs, aorta, thoracic spinal cord, tongue, trachea, esophagus, thyroid gland, eyes, brain, pituitary gland, and skin (including mammary glands).

Histopathologic examinations were conducted for all rats in the control and high-dose groups, as well as for organs with gross abnormalities identified during necropsy in the low and mid-dose groups. The organs or tissues were dehydrated, embedded in paraffin, sectioned at 4–5 µm thickness, and stained with Hematoxylin and Eosin. The scope of histopathological examination was prespecified in accordance with Notification No. 2005-60 issued by the KFDA, now the Korean MFDS. This notification specifies examination of the control and high-dose groups, with examination of other dose groups when warranted by the findings. Since no histopathological changes attributable to the SBE were observed in the control and high-dose groups, histopathological examinations were not extended to the other lower dose groups.

Histopathological findings were recorded for individual animals by organ. Lesion incidence was summarized as the number and percentage of affected animals among those examined, separately by sex and group. Lesion severity was graded as 1+ (minimal), 2+ (mild), 3+ (moderate), or 4+ (marked).

### 2.5. Statistical Analysis

Data from male and female rats were analyzed separately. Body weight and food and water consumption were compared between groups separately at each measurement time point. No repeated-measures analysis was performed. Continuous data were analyzed using one-way analysis of variance (ANOVA). When ANOVA indicated a statistically significant difference, post hoc comparisons with the control group were performed using tests selected based on group size and the results of Levene’s test for homogeneity of variances. When variances were homogeneous and group sizes were equal, Duncan’s multiple range test was used for pairwise comparisons among group means [18]. When variances were homogeneous but group sizes were unequal, Scheffé’s procedure was used because it accommodates unequal sample sizes and provides simultaneous inference for all contrasts among group means [19]. When the equal-variance assumption was not met, Dunnett’s T3 test was used for pairwise comparisons because it does not require a common variance across groups [20]. Discrete data were transformed before applying the Kruskal–Wallis H-test. If significance was identified, the Mann–Whitney U-test was employed to confirm significance with the control group. Fisher’s exact test was applied to urine color and the incidences of individual necropsy and histopathological findings. Data were expressed as mean ± standard deviation (SD). A *p*-value of <0.05 was considered statistically significant. SPSS (version 10.1, SPSS Inc., Chicago, IL, USA) was used for statistical analysis.

## 3. Results

### 3.1. Acute Oral Toxicity Study

No deaths were observed in any of the groups during the study period. On the day of administration, one female rat in the 2500 mg/kg dose group exhibited soft stool, while in the 5000 mg/kg dose group, soft stool and soiled perineal regions were observed in one male and three female rats, respectively. These symptoms had disappeared by the first day following administration. Body weight changes and gains observed over the 14 days following administration were similar across all groups for both sexes (Table 1). Necropsy findings included testicular and epididymal atrophy in one male rat from the 5000 mg/kg dose group, with the organs displaced to the right upper abdomen and adhered to the diaphragm. Additionally, one female rat from both the control group and the 5000 mg/kg dose group exhibited uterine retention of clear liquid.

### 3.2. Twenty-Six-Week Repeated-Dose Oral Toxicity Study

#### 3.2.1. Clinical Signs

During the experimental period, no deaths or clinical symptoms were observed in any of the groups, including both the main and recovery groups.

#### 3.2.2. Body Weight

**Figure 1:**
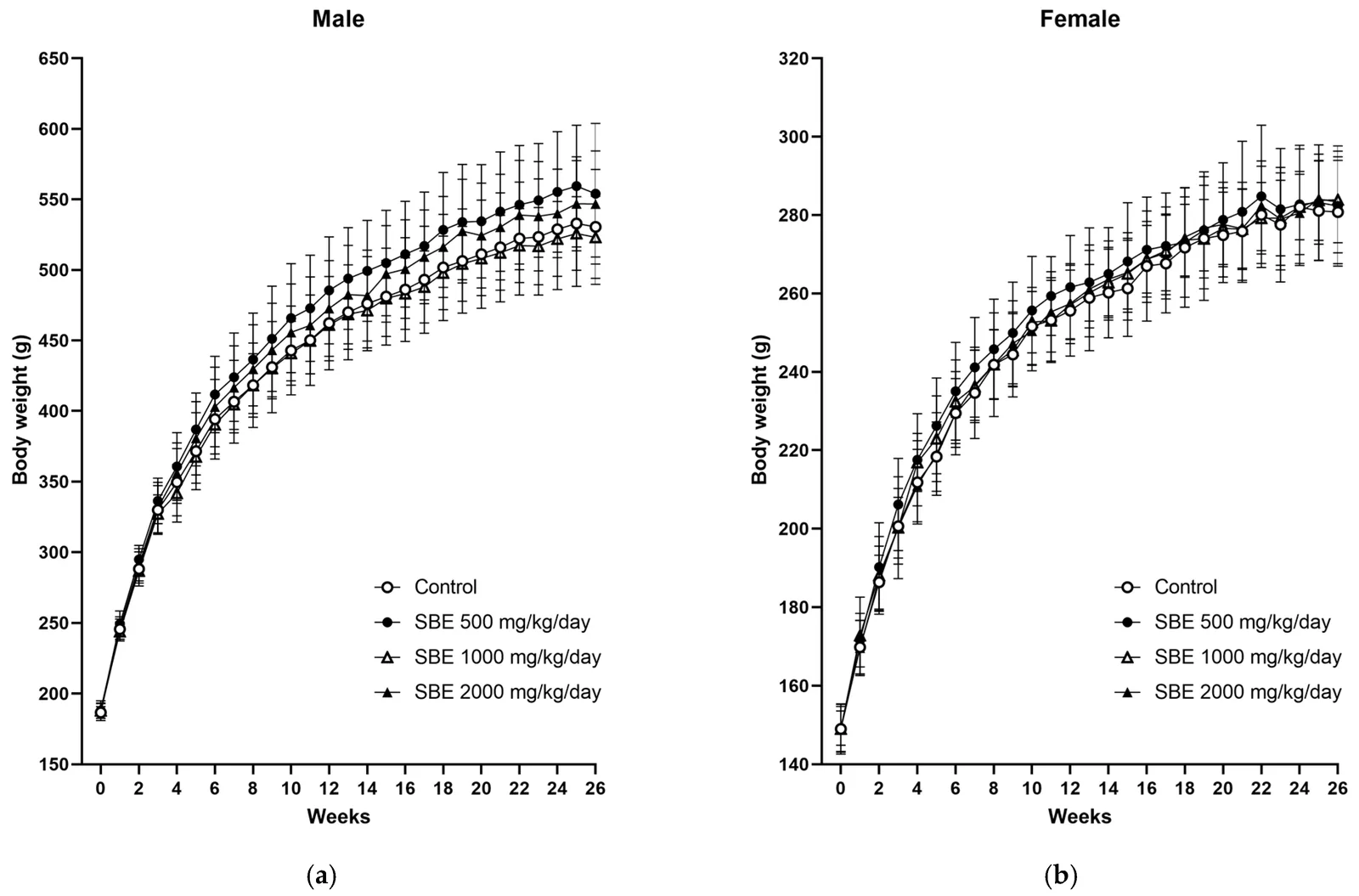

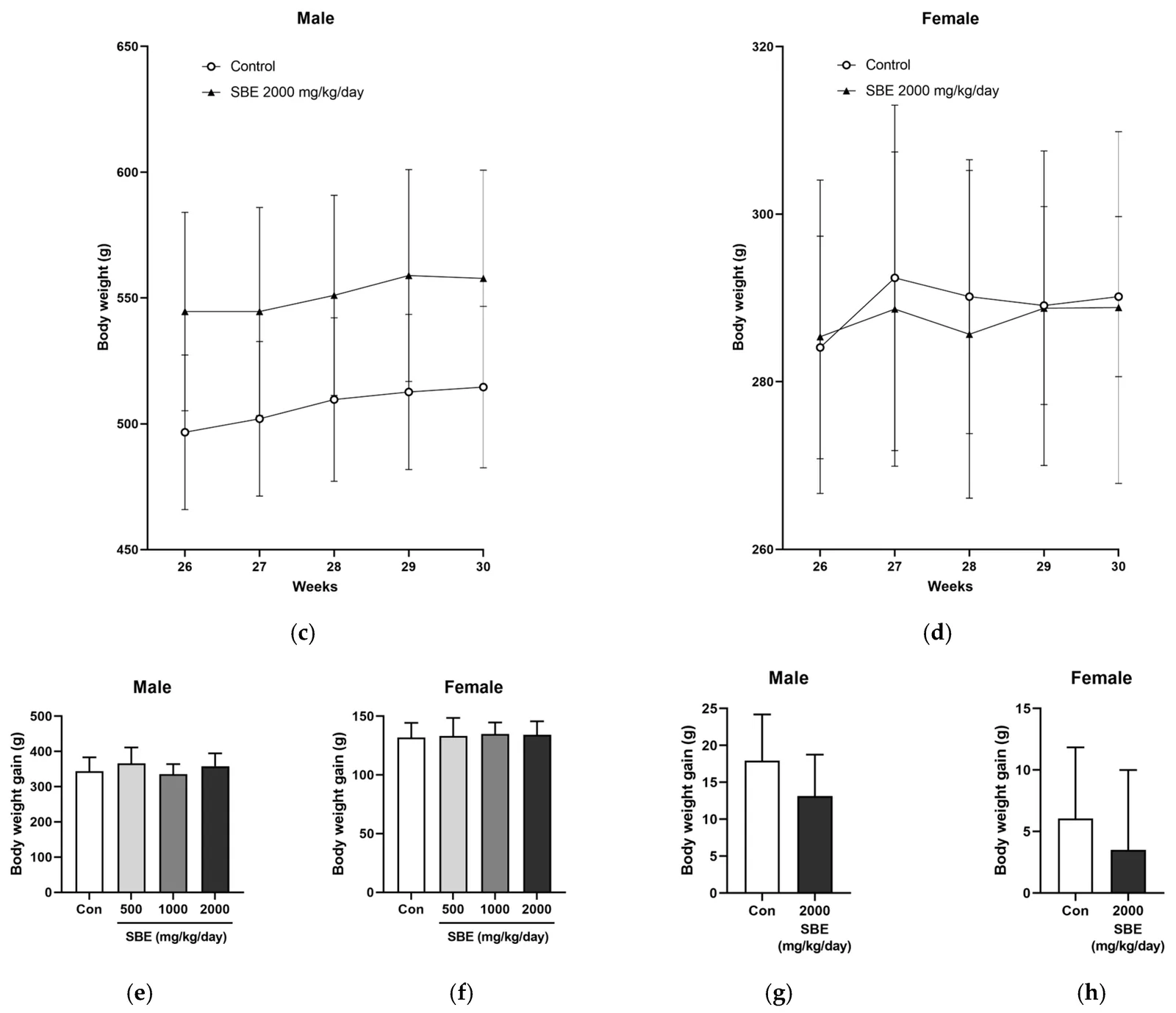
Body weight changes and weight gains of male and female rats during the administration and recovery period in the 26-week repeated-dose oral toxicity study of *Scutellaria baicalensis*. (**a**,**b**) represent the body weights of male and female rats, respectively, during the administration period, while (**c**,**d**) represent the body weights of male and female rats, respectively, during the recovery period. (**e**,**f**) show the body weight gains of male and female rats, respectively, over the 26-week period, while (**g**,**h**) show the body weight gains of male and female rats, respectively, during the 4-week recovery period. Con, control; SBE, *Scutellaria baicalensis* extract. Values are mean ± SD.

During the 26-week administration period, males in the 500 and 2000 mg/kg/day dose groups tended to show increased body weights and weight gains compared to the control group (Figure 1a,e). Nonetheless, none of these trends were statistically significant. For females, no significant differences in body weight were observed between the groups during both the administration and recovery periods.

#### 3.2.3. Food and Water Consumption

**Figure 2:**
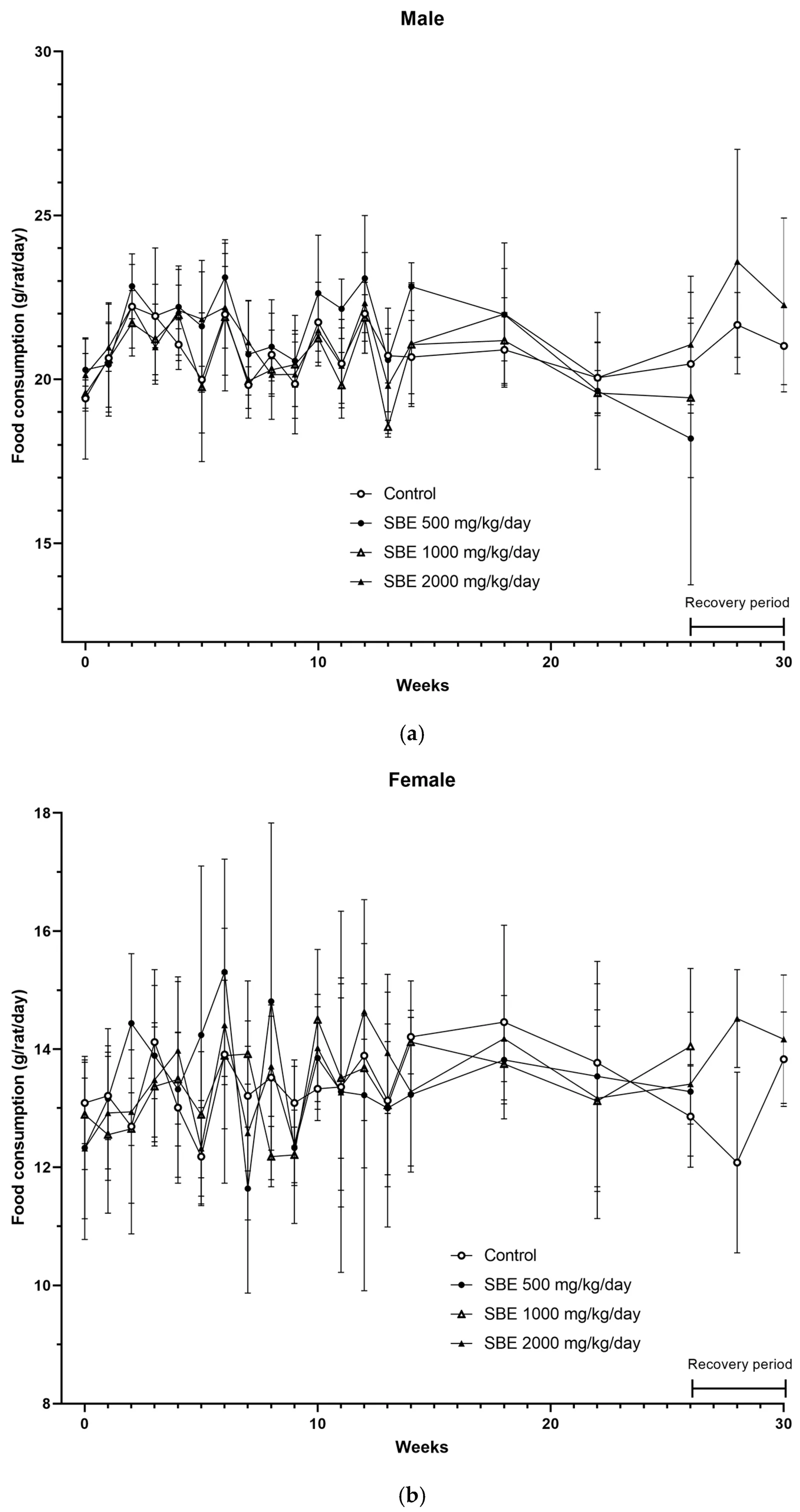
Food consumption of male (**a**) and female (**b**) rats in the 26-week repeated-dose oral toxicity study of *Scutellaria baicalensis* extract (SBE). Values are mean ± SD.

**Figure 3:**
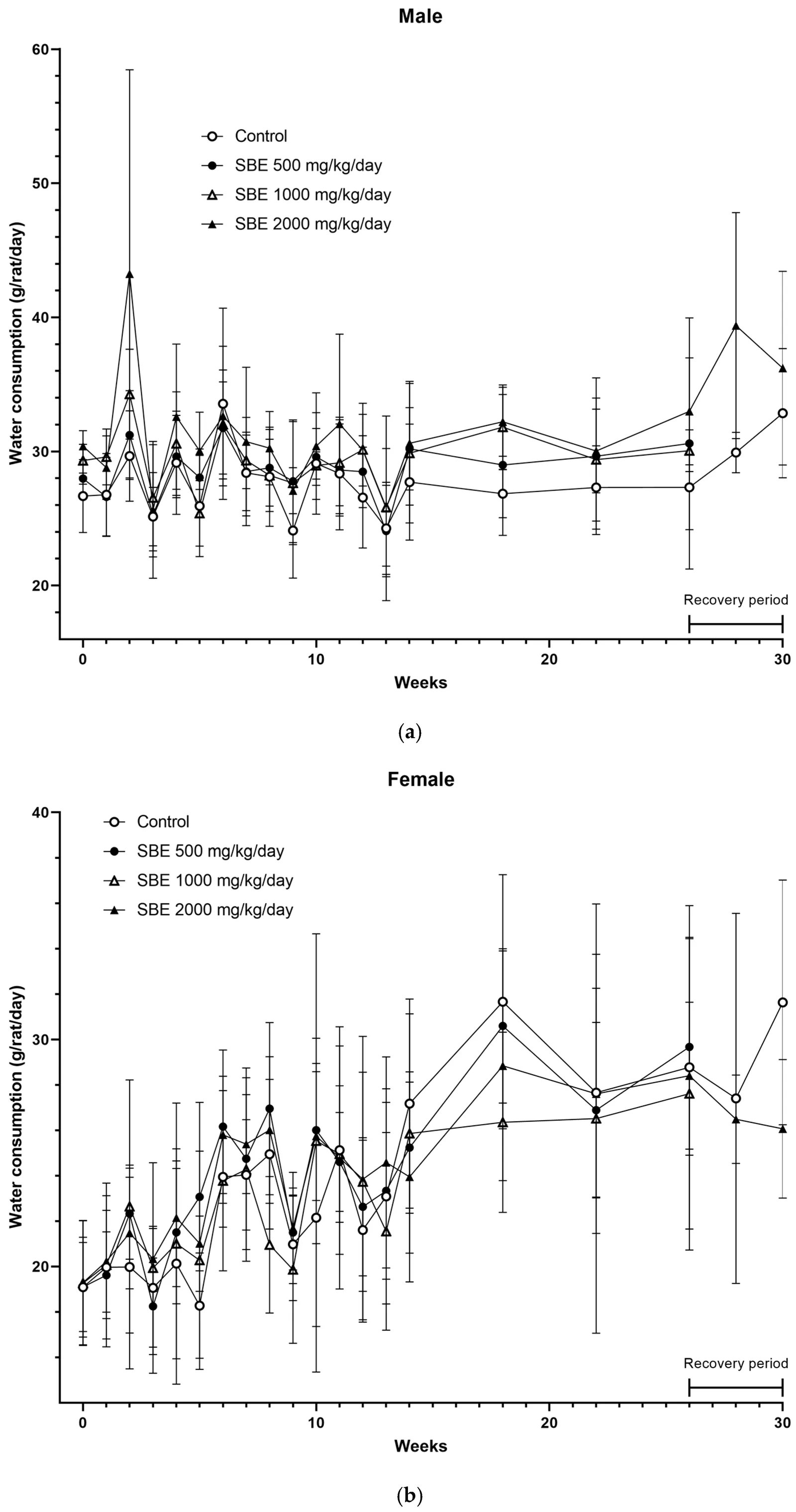
Water consumption of male (**a**) and female (**b**) rats in the 26-week repeated-dose oral toxicity study of *Scutellaria baicalensis* extract (SBE). Values are mean ± SD.

There was no significant change in food consumption across all treatment and control groups for both male and female rats (Figure 2). Males in all SBE dosing groups exhibited a tendency toward increased water consumption compared to the control group throughout the administration and recovery periods; however, these increases were not statistically significant (Figure 3a). Water consumption among female rats did not show any significant differences between the groups during the experimental period (Figure 3b).

#### 3.2.4. Ophthalmologic Examination

External eye and fundus examinations revealed no ophthalmologic abnormalities in either sex in the control or high-dose groups.

#### 3.2.5. Urinalysis

Ketone body grades shifted toward higher categories with increasing dose during the administration period in both male and female rats, with significant increases (*p* < 0.05) observed in both sexes at a dose of 2000 mg/kg/day compared to the control group (Table 2). At 2000 mg/kg/day, 1 + ketones were observed in 3/5 males and 2/5 females, compared with 0/5 animals in each control group; the remaining high-dose animals had trace ketones. In the recovery groups, no statistically significant differences in ketone body levels were observed between the SBE and control groups in either sex.

The following numerical differences were observed in the parameters of specific gravity, pH, protein, nitrite, leukocyte, and occult blood, although none of these reached statistical significance: (1) specific gravity tended toward higher categories at 1000 and 2000 mg/kg/day in both sexes (values ≥ 1.020 in 5/5 animals of each sex at 2000 mg/kg/day, compared with 3/5 males and 1/5 females in the control groups), (2) pH levels exhibited a decreasing trend in males at doses of 1000 and 2000 mg/kg/day, (3) protein, nitrite, and leukocyte levels showed an increasing trend in females at doses of 1000 and 2000 mg/kg/day, and (4) occult blood levels tended to decrease in the SBE-treated groups in both sexes. Urine sediment examination results showed no dose-related changes in either the main or recovery groups in both sexes.

Urine volume decreased numerically across doses in female main groups, with a 37.67% lower mean at 2000 mg/kg/day than in controls (18.2 ± 3.7 vs. 29.2 ± 13.5 mL). In the female recovery groups, urine volume was also numerically lower at 2000 mg/kg/day (control 24.4 ± 7.7 mL, SBE 2000 mg/kg/day 18.4 ± 7.2 mL; 24.59% lower), with no statistically significant differences in either the main or recovery groups.

The frequency of dark yellow urine increased with dose in both sexes during the administration period, with a significant increase (*p* < 0.01) observed in females at a dose of 2000 mg/kg/day compared to the control group (5/5 vs. 0/5 rats). However, no difference in urine color was observed during the recovery period between the SBE and control groups.

#### 3.2.6. Hematology

The WBC counts in males receiving doses of 500 and 2000 mg/kg/day significantly increased by 34.67% and 32.64%, respectively, compared to the control group (*p* < 0.01), although the increase did not show dose-dependency (Table 3). After the recovery period, the WBC counts in males treated with SBE at 2000 mg/kg/day (8.52 ± 0.93 × 10^3^/μL) were numerically 8.59% lower than those in the control group (9.32 ± 1.76 × 10^3^/μL), with no significant difference noted. Neutrophil percentages were numerically higher in all SBE-treated male groups; however, this increase was not statistically significant. Monocyte percentages were numerically lower in males at 1000 and 2000 mg/kg/day and in all SBE-treated female groups, with a significant decrease of 18.03% observed in male rats at a dose of 2000 mg/kg/day compared to the control group (*p* < 0.05). After the recovery period, monocyte percentages in SBE-treated males at 2000 mg/kg/day (3.5 ± 0.7%) were numerically 10.19% higher than those in the control group (3.1 ± 0.6%), yet no significant difference was observed.

#### 3.2.7. Serum Biochemistry

A significant increase of 28.65% in triglyceride levels was observed in females at a dose of 2000 mg/kg/day compared to the control group (*p* < 0.05, Table 4). After the recovery period, triglyceride levels were numerically higher in the 2000 mg/kg/day male recovery group than in the control group (117.0 ± 21.7 vs. 83.6 ± 29.6 mg/dL; 39.95% higher), but the difference was not statistically significant.

Albumin levels were numerically lower in all SBE-treated groups of both sexes, with decreases of 2.84% in males and 3.32% in females at 2000 mg/kg/day, but no significant difference was found compared to the control group. Creatinine levels in females and Ca^2+^ levels in both sexes were also numerically lower in the SBE-treated groups than in the control group, without statistically significant differences.

#### 3.2.8. Plasma Coagulation

In females of the main groups, aPTT exhibited a dose-dependent increasing trend, but it did not reach a significant difference compared to the control group (Table 5). In the recovery groups, prothrombin time and aPTT were similar between the control and 2000 mg/kg/day groups (males: 15.80 ± 0.66 vs. 15.66 ± 0.48 s and 17.96 ± 0.60 vs. 17.84 ± 0.98 s; females: 16.76 ± 0.22 vs. 16.66 ± 0.26 s and 18.64 ± 1.57 vs. 18.74 ± 0.91 s, respectively).

#### 3.2.9. Organ Weight

The absolute and relative liver weights were numerically higher in the SBE-treated groups in both male and female rats, with significant increases in both the absolute (12.79%) and relative (12.50%) weights observed in the 2000 mg/kg/day female group (*p* < 0.01 and *p* < 0.05, respectively; Table 6) and in all SBE-treated male groups’ relative weights (9.31%, 9.16%, and 14.97% at 500, 1000, and 2000 mg/kg/day, respectively; all *p* < 0.05) compared to the control group. After the recovery period, an increasing trend in liver weight was observed in the SBE 2000 mg/kg/day-treated male recovery group (absolute weight: 13.5458 ± 1.2001 g; relative weight: 2.5492 ± 0.0738% of body weight), but this was not statistically significant when compared to the control group (absolute weight: 11.2826 ± 1.6751 g; relative weight: 2.2986 ± 0.2152% of body weight). In males, both the absolute and relative weights of the spleen and the relative weights of the left and right kidneys showed an increasing trend in the treated groups, but these were not significantly different from the control. A significant increase of 18.09% in the relative weight of the left adrenal gland was observed in males treated with 500 mg/kg/day (*p* < 0.05) compared to the control group, but this change did not correlate with dose.

#### 3.2.10. Necropsy

Upon necropsy of the main groups, small thymuses were observed in one case each in the male and female control groups, as well as in the male 1000 mg/kg/day group. Diffuse red spots in the thymus were observed in one case each in the male control group and the female 1000 mg/kg/day group. Red mandibular lymph nodes were observed in two cases in the male 2000 mg/kg/day group and in one case in the female 1000 mg/kg/day group. Red sublingual lymph nodes were observed in two cases in the male 2000 mg/kg/day group. A red ovary was observed in one case in the female 2000 mg/kg/day group. Necropsy of the uterus in the female main groups revealed a cyst in one case in the 500 mg/kg/day group, and retention of clear fluid was observed in one case each in the control and 500 mg/kg/day groups, and in three cases in the 1000 mg/kg/day group. In the female recovery groups, a cyst filled with clear liquid in the ovary and a milky round spot in the kidney were each observed in one case in the control group. Additionally, retention of clear fluid in the uterus was observed in one case in the female 2000 mg/kg/day recovery group.

#### 3.2.11. Histopathology

Organs observed with findings in both males and females included the kidneys, lungs, mandibular lymph nodes, thymus, and thyroid, with specific findings, incidences, and severity grades detailed in Table S1 and Table S2. There were no statistically significant differences in the incidence of the listed histopathological findings between the control and 2000 mg/kg/day groups in either sex in the main or recovery groups.

## 4. Discussion

This study aimed to assess the potential toxicity of SBE after single and 26-week re-peated oral administration in rats of both sexes. No adverse effects were identified at the tested doses, although some treatment-related changes were observed during repeated administration.

In the acute toxicity study, single oral administration of SBE at doses up to 5000 mg/kg caused no mortality or treatment-related changes in body weight or gross necropsy findings. Transient soft stools and soiled perineal regions were considered treatment-related but non-adverse clinical signs. Consistent with these findings, previous single-dose studies of aqueous, hydroalcoholic, and fermented extracts of Scutellariae Radix similarly reported no mortality or treatment-related toxicity at the tested doses [8,9,10]. Under the present experimental conditions, the minimum lethal dose (MLD) of SBE was considered to exceed 5000 mg/kg in rats of both sexes, indicating low acute oral toxicity. This interpretation was based on the absence of mortality at the highest tested dose; the exact MLD was not determined. Based on the U.S. Environmental Protection Agency acute oral toxicity classification, this level corresponds to Toxicity Category IV, the lowest toxicity category, for which no acute oral hazard statement is required [21].

During the 26-week administration period, no mortality or treatment-related clinical abnormalities were observed at doses up to 2000 mg/kg/day. Body weights tended to in-crease in males receiving 500 and 2000 mg/kg/day, although the changes were not statistically significant. No toxicologically relevant changes were observed in food or water consumption or ophthalmologic examinations. All necropsy and histopathological findings were neither dose-dependent nor statistically significant and were considered spontaneous or sporadic lesions [22,23]; thus, they were considered unrelated to SBE administration. Yi et al. (2018) similarly reported no mortality or significant changes in body weight or food consumption during 26 weeks of SBE administration at doses up to 2500 mg/kg/day, and no gross pathological abnormalities were observed [13].

At the end of the 26-week administration period, statistically significant differences from the controls were identified in eight parameters: (1) WBC counts increased in males at 500 and 2000 mg/kg/day; (2) monocyte percentage decreased in males at 2000 mg/kg/day; (3) relative left adrenal weight increased in males at 500 mg/kg/day; (4) the incidence of dark yellow urine increased in females at 2000 mg/kg/day; (5) urinary ketone grades increased in both sexes at 2000 mg/kg/day; (6) serum triglyceride levels increased in females at 2000 mg/kg/day; (7) absolute liver weight increased in females at 2000 mg/kg/day; and (8) relative liver weight increased in males at 500, 1000, and 2000 mg/kg/day and in females at 2000 mg/kg/day. The magnitude and dose–response pattern of each change, published control data, and related clinical and pathological findings were considered to assess treatment relatedness and adversity.

The increases in WBC counts and decrease in monocyte percentage were not clearly related to SBE administration. In males, the WBC increases did not show a monotonic dose–response relationship, and the mean counts at 500 and 2000 mg/kg/day (9.77 and 9.62 × 10^3^/μL, respectively) were within the range reported in published control data for male Crl:CD(SD) rats aged 23–47 weeks (9.40–10.10 × 10^3^/μL) [24]. The decrease in monocyte percentage at 2000 mg/kg/day occurred alongside an increased total WBC count and did not necessarily indicate a reduction in absolute monocyte numbers. The mean monocyte percentage of 3.4% was also comparable to control means for male SD rats reported in the 26-week PM012 and UP446 studies (3.05% and 3.7%, respectively) [25,26]. In particular, the PM012 study used the same animal supplier and hematology analyzer as the present study. Furthermore, these hematological changes were not accompanied by corresponding clinical signs or treatment-related histopathological findings in the present study. Consistent with this interpretation, Yi et al. reported no significant differences in WBC counts or monocyte percentages in either sex after 26 weeks of administration of an ethanol extract of *S. baicalensis* to Wistar rats at doses up to 2500 mg/kg/day [13].

Relative left adrenal weight in males was significantly increased only at 500 mg/kg/day and was comparable to the control value at 2000 mg/kg/day. In addition, the relative left adrenal weight at 500 mg/kg/day (0.0058 ± 0.0008%) was numerically similar to the male control value reported in the 26-week PM012 study (0.0054 ± 0.0007%), which used male SD rats from the same supplier [25]. Taken together, these findings supported the interpretation that the increase was incidental and unlikely to be related to SBE administration.

The increased incidence of dark yellow urine in high-dose females was considered related to SBE administration, given the dose-associated increase in both sexes. In females, the numerically lower urine volume at 2000 mg/kg/day than in controls (18.2 ± 3.7 vs. 29.2 ± 13.5 mL) and the greater proportion of animals with a urine specific gravity ≥ 1.020 (5/5 vs. 1/5) suggested that urine concentration may have contributed to the darker color. Yi et al. similarly reported dose-associated darkening of urine following administration of an ethanol extract of *S. baicalensis* at doses up to 2500 mg/kg/day and suggested a relationship with the color of the extract [13]. The urine discoloration was not accompanied by serum biochemical or histopathological evidence of treatment-related hepatic or renal injury. Dark yellow urine was not observed in the recovery groups. The discoloration was therefore considered non-adverse.

The liver-weight findings were evaluated together with the changes in urinary ketones and serum triglycerides. Relative liver weights were significantly increased in males at all three doses, whereas both absolute and relative liver weights were significantly increased in females at 2000 mg/kg/day. The mean relative liver weights in males at 500 and 1000 mg/kg/day (2.5531% and 2.5494%, respectively; Table S3) were comparable to control means for male SD rats reported in the 26-week PM012 and UP446 studies (2.4422% and 2.53%, respectively) [25,26] and were also within the reference range reported in the test facility’s historical control data for 32-week-old male SD rats (2.0312–2.8946% of body weight; *n* = 50). Body weight differences were also considered when interpreting the male findings: at 1000 mg/kg/day, mean terminal body weight was 5.82% lower than the control value, while absolute liver weight was only 3.53% higher (Table S3), suggesting that the lower body weight contributed in part to the increased organ-to-body weight ratio. The relative liver-weight increases at 500 and 1000 mg/kg/day, without accompanying significant increases in absolute liver weight or significant changes in serum chemistry or urinary ketone grades, were interpreted as incidental variation when considered together with the published and historical control data and the possible contribution of lower terminal body weight at 1000 mg/kg/day. These lower-dose findings were therefore not considered treatment-related. At 2000 mg/kg/day, the liver-weight increases coincided with increased urinary ketone grades in both sexes and increased serum triglycerides in females. This combined pattern, observed only at 2000 mg/kg/day, supported an SBE-related hepatic and metabolic response. However, these findings were not accompanied by significant increases in conventional serum markers of hepatic injury or treatment-related hepatic histopathological lesions. The nonsignificant decrease in serum albumin was not accompanied by a corresponding decrease in total protein and did not, by itself, establish impaired hepatic protein synthesis. Yi et al. also reported significant increases in relative liver weight in female Wistar rats receiving a 70% ethanol extract of Scutellariae Radix at 300 and 1250 mg/kg/day for 26 weeks, without significant increases in serum biochemical indicators of hepatocellular injury and cholestasis and without notable hepatic histopathological changes [13]. Based on this integrated assessment, the hepatic and metabolic changes observed at 2000 mg/kg/day were considered treatment-related but non-adverse under the present experimental conditions.

Several mechanisms may account for the liver-weight increases, including enzyme-induction-associated hepatocellular hypertrophy, altered intracellular lipid or glycogen storage, and proliferative, inflammatory, congestive, or hydropic changes [27]. The absence of treatment-related hepatic histopathological lesions at 2000 mg/kg/day did not support the latter processes as primary explanations. Previous studies have reported that Scutellariae Radix extracts increased hepatic CYP1A and glutathione S-transferase Yc1/2 expression in rats and CYP2E1-mediated metabolism in humans, while baicalein induced CYP3A4 expression in vitro [28,29,30,31]. These enzyme-related findings provide indirect support for an adaptive hepatic response involving induction of selected drug-metabolizing and detoxification enzymes as a possible explanation. Altered glycogen storage is another possibility: baicalin significantly increased hepatic glycogen in diabetic rats [32], whereas Scutellariae Radix extract tended to increase hepatic glycogen in rats [33]. These glycogen findings suggest altered hepatic glycogen storage as a possible explanation.

Urine concentration and altered ketone metabolism are possible contributors to the increased urinary ketone grades. At 2000 mg/kg/day, urine volume was numerically lower and a greater proportion of rats had a urine specific gravity ≥ 1.020 in both sexes compared with controls, although neither difference was statistically significant. The reagent-strip assay (Multistix 10SG, Bayer, USA) used in the present study provides a semiquantitative measure of urinary acetoacetate concentration. Consequently, more concentrated urine could yield higher ketone grades even if the total amount of acetoacetate excreted remained unchanged. A metabolic contribution is also plausible: Dai et al. demonstrated that baicalin activated hepatic carnitine palmitoyltransferase 1A (CPT1A), enhanced fatty acid oxidation, and increased plasma total ketone bodies in diet-induced obese mice [34]. Li et al. reported that *S. baicalensis* root extract counteracted the reduction in urinary β-hydroxybutyrate, one of the ketone bodies, in D-galactose-induced aging rats [35]. These findings suggest a possible contribution of altered ketone metabolism, although differences in experimental models, sample matrices, and measured ketone species limit direct comparison.

The triglyceride increase may reflect altered lipid metabolism. Consistent with our finding, Yi et al. reported increased serum triglycerides after 26 weeks of oral administration of an ethanol extract of *S. baicalensis*, with normalization after a 4-week recovery period [13]. Triglyceride elevations were also reported in single- and multiple-dose phase I trials of baicalein in healthy volunteers [36,37]. The increase observed in the present study could result from increased hepatic very-low-density lipoprotein (VLDL) secretion or reduced peripheral triglyceride clearance. Regarding the latter possibility, baicalin and baicalein have been reported to reduce lipoprotein lipase (LPL) expression in cell models of adipogenic differentiation [38,39]. Because LPL participates in the hydrolysis of circulating triglycerides, these findings provide an indirect basis for considering reduced triglyceride clearance. However, all adaptive and metabolic mechanisms proposed in this Discussion remain hypothetical because they were not directly investigated in the present study.

Potential renal effects were also considered because baicalin, a major flavonoid of Scutellariae Radix, has been reported to induce renal injury and fibrosis in rats at doses of 400–1600 mg/kg/day [14]. In the present study, urinary specific gravity increased or tend-ed to increase, while urinary protein, nitrite, and leukocytes tended to increase and urine volume tended to decrease in females. Serum creatinine tended to decrease in females, neutrophil percentages tended to increase in males, and relative kidney weights tended to increase in males. However, these changes were not statistically significant or consistently supported by other renal parameters, and no treatment-related renal histopathological lesions were observed. Therefore, these findings were not considered indicative of a treatment-related renal effect. More recently, administration of baicalin at 4000 mg/kg/day for 28 days in mice was associated with increased serum creatinine in females and mild renal inflammatory changes [16]. However, direct comparison is limited because the previous studies evaluated baicalin itself, whereas baicalin constituted 19.8% of the SBE used in the present study. No adverse renal effects were identified following SBE administration at doses up to 2000 mg/kg/day in the present study.

Cardiac injury has also been reported following intravenous administration of wogonin at 120 mg/kg/day, another major flavonoid of Scutellariae Radix [15]. In the present study, however, no consistent treatment-related changes in heart weight, gross pathology, or histopathology were observed. A single case of focal myocarditis in a high-dose male was considered spontaneous because it occurred in only one animal and was not accompanied by other related findings. Thus, the heart was not considered a target organ under the present experimental conditions.

Despite the absence of hepatotoxicity in the present study, clinical cases of *S. baicalensis*-associated liver injury have been reported. Sporadic cases of clinically apparent liver injury have been associated with the use of *S. baicalensis*-containing products [11], and a recent review identified 16 suspected cases of *S. baicalensis*-associated drug-induced liver injury [12]. These cases have been suggested to represent rare idiosyncratic reactions, potentially involving immune-mediated mechanisms. Conventional repeated-dose toxicity studies in healthy animals are primarily designed to identify reproducible, dose-related toxicity and may not detect rare idiosyncratic reactions. Therefore, the absence of treatment-related hepatic lesions in the present study does not exclude the possibility of idiosyncratic liver injury in susceptible individuals.

Findings after the 4-week recovery period further supported the reversibility of the observed effects. Urinary ketone bodies in males showed a decreasing trend toward control levels, while most other parameters were comparable between control and SBE-treated animals. Although relative liver weight in males remained numerically higher and urine volume in females remained lower, neither difference was statistically significant. Overall, the treatment-related changes observed during administration tended to return toward control levels.

The NOEL and NOAEL represent the highest tested doses without observed treatment-related effects and without observed treatment-related adverse effects, respectively. Treatment relatedness and adversity were evaluated through an integrated assessment that considered not only statistical significance but also the magnitude and dose pattern of changes and related study findings [40,41]. Based on this integrated assessment, no findings at doses up to 1000 mg/kg/day were judged to be treatment-related; therefore, the NOEL was determined to be 1000 mg/kg/day. At 2000 mg/kg/day, treatment-related but non-adverse findings included statistically significant increases in relative liver weight and urinary ketone grades in both sexes, as well as in absolute liver weight, serum triglyceride levels, and the incidence of dark yellow urine in females. These findings were considered non-adverse because they were not accompanied by corroborating clinical, serum biochemical, or histopathological evidence of organ injury. Therefore, 2000 mg/kg/day was established as the NOAEL, and no target organ of toxicity was identified.

The rat NOAEL of 2000 mg/kg/day corresponds to an estimated human-equivalent extract dose of approximately 324.3 mg/kg/day based on body-surface-area scaling (2000 × 6/37, with rat and adult human K_m_ factors of 6 and 37, respectively) [42]. For a 60-kg adult, this corresponds to approximately 19.46 g of extract per day and, based on the extraction yield of 26.08%, approximately 75 g/day of Scutellariae Radix raw material (19.46/0.2608). This raw-material-equivalent amount is approximately 7.5–25 times the daily raw-material dose of 3–10 g described in the Chinese Pharmacopoeia. However, this calculation does not account for differences in constituent composition among preparations or interspecies differences in pharmacokinetics, including absorption, metabolism, and elimination. It therefore serves only as a theoretical dose comparison and does not establish equivalent systemic exposure.

One limitation of the present study is that the highest repeated dose was 2000 mg/kg/day, which was lower than the 2500 mg/kg/day dose associated with hepatic inflammatory changes in the previous 26-week study published in 2018 [13]. Thus, potential effects at higher doses cannot be excluded. The histopathological examination plan was consistent with the approach described in OECD TG 408, which recommends full examination of the control and high-dose groups and extension to lower-dose groups when treatment-related histopathological changes are observed in the high-dose group [43,44]. Nevertheless, histopathological examination was not systematically performed in the 500 and 1000 mg/kg/day groups, limiting the assessment of potential dose-dependent histopathological changes. In particular, the relationship between liver weight increases and microscopic hepatic changes could not be fully assessed across dose groups, although no treatment-related hepatic histopathological lesions were observed in either sex at 2000 mg/kg/day. Overall, the present GLP-compliant study provides comprehensive information on the acute and long-term repeated-dose toxicity of SBE, including its MLD, NOEL, NOAEL, reversibility, and potential target-organ profile.

## 5. Conclusions

In conclusion, single oral administration of SBE caused no mortality at doses up to 5000 mg/kg, and the MLD was considered to exceed 5000 mg/kg in rats of both sexes. In the 26-week repeated-dose study, treatment-related findings at 2000 mg/kg/day included increased liver weight, together with urinalysis and serum biochemical changes suggestive of hepatic effects. These changes were considered non-adverse and tended to return toward control levels after the 4-week recovery period. The NOEL and NOAEL were therefore determined to be 1000 and 2000 mg/kg/day, respectively, and no target organ of toxicity was identified. These findings provide comprehensive preclinical safety information for SBE following acute and long-term repeated oral exposure.

## Figures and Tables

**Table 1:** Body weight changes and gains in the acute toxicity study of *Scutellaria baicalensis* extract.

Groups	Day 0 ^1^	Day 1	Day 3	Day 7	Day 14	Gains
**Male**						
Control	226.20 ± 2.81 ^2^	253.46 ± 3.81	267.59 ± 6.00	294.77 ± 8.58	337.25 ± 13.01	111.06 ± 10.90
2500 mg/kg	227.76 ± 4.49	254.89 ± 5.31	269.25 ± 8.83	293.28 ± 9.25	325.02 ± 14.59	97.26 ± 12.14
5000 mg/kg	227.53 ± 4.72	252.92 ± 5.31	267.13 ± 5.73	291.44 ± 5.52	328.92 ± 7.98	101.39 ± 8.90
**Female**						
Control	172.49 ± 2.29	193.49 ± 2.69	197.26 ± 4.74	202.88 ± 3.81	217.01 ± 4.46	44.52 ± 4.47
2500 mg/kg	175.14 ± 2.28	192.81 ± 4.42	197.07 ± 3.89	203.00 ± 6.27	213.74 ± 9.04	38.60 ± 7.84
5000 mg/kg	173.72 ± 3.78	193.17 ± 3.06	198.37 ± 5.22	202.41 ± 5.20	216.55 ± 7.97	42.83 ± 7.78

^1^ The day of administration was designated Day 0. ^2^ Data are expressed as mean ± SD in grams (g). Male and female data are presented in the upper and lower sections, respectively, with bold subheadings distinguishing the two sections.

**Table 2:** Summary of selected ^1^ urinalysis findings in the 26-week repeated-dose oral toxicity study of *Scutellaria baicalensis* extract.

Parameter	Result	Groups (mg/kg/Day)							
		Male				Female			
		0	500	1000	2000	0	500	1000	2000
Ketone body	−	4	1	1	0	5	5	2	0
	+/−	1	4	3	2	0	0	3	3
	1+	0	0	1	3 *	0	0	0	2 *
Specific gravity	≤1.005	0	0	0	0	0	2	0	0
	1.01	2	1	0	0	0	0	0	0
	1.015	0	1	1	0	4	2	1	0
	1.02	2	2	2	3	0	1	1	2
	1.025	1	1	2	2	1	0	2	2
	≥1.030	0	0	0	0	0	0	1	1
pH	6.5	0	0	0	0	0	0	0	0
	7.0	0	0	0	0	0	0	0	0
	7.5	1	0	0	0	0	0	1	0
	8.0	1	2	4	5	5	2	3	2
	8.5	3	3	1	0	0	3	1	3
	≥9.0	0	0	0	0	0	0	0	0
Protein	−	0	0	0	0	3	4	1	0
	+/−	0	0	0	0	0	1	0	0
	1+	0	1	1	0	1	0	2	0
	2+	1	0	1	1	1	0	1	1
	3+	4	4	3	4	0	0	1	4
Nitrite	−	5	5	5	5	5	5	4	2
	+	0	0	0	0	0	0	1	3
Occult blood	−	1	5	5	4	3	5	5	5
	+/−	2	0	0	1	2	0	0	0
	1+	2	0	0	0	0	0	0	0
Leukocyte	−	0	0	0	0	3	4	1	0
	+/−	0	0	0	0	1	1	2	0
	1+	1	1	0	0	1	0	1	3
	2+	4	0	2	4	0	0	1	2
	3+	0	4	3	1	0	0	0	0
Urine volume (mL)		17.4 ± 4.7 ^2^	14.6 ± 1.1	18.8 ± 7.1	15.4 ± 5.7	29.2 ± 13.5	26.0 ± 7.3	20.2 ± 6.4	18.2 ± 3.7
Urine color	Yellow	5	5	3	2	5	5	2	0
	Dark Yellow	0	0	2	3	0	0	3	5 **
No. of rats examined		5	5	5	5	5	5	5	5

^1^ Only parameters with statistical significance or tendency are shown in table. ^2^ Data are expressed as mean ± SD. * *p* < 0.05 and ** *p* < 0.01 compared with the control. Bold values indicate statistical significance.

**Table 3:** Summary of selected ^1^ hematological findings in the 26-week repeated-dose oral toxicity study of *Scutellaria baicalensis* extract.

Parameter (Units)	Groups (mg/kg/Day)							
	Male				Female			
	0	500	1000	2000	0	500	1000	2000
WBC (×10^3^/μL)	7.25 ± 0.89 ^2^	9.77 ± 1.62 **	8.58 ± 2.11	9.62 ± 1.02 **	3.98 ± 1.03	4.23 ± 1.02	4.49 ± 1.10	4.87 ± 1.48
Differential leukocyte counts								
Neutrophils (%)	26.8 ± 8.2	31.5 ± 10.4	30.5 ± 14.3	37.1 ± 11.6	17.0 ± 5.0	17.7 ± 6.2	17.5 ± 4.5	17.4 ± 4.7
Lymphocytes (%)	66.4 ± 7.4	61.3 ± 10.1	63.1 ± 13.8	56.8 ± 10.7	75.5 ± 5.8	76.3 ± 5.6	76.3 ± 4.2	75.8 ± 4.3
Monocytes (%)	4.2 ± 0.9	4.4 ± 0.8	3.8 ± 0.7	3.4 ± 0.6 *	3.8 ± 1.0	3.1 ± 0.8	3.2 ± 0.9	3.4 ± 0.8
Eosinophil (%)	1.2 ± 0.3	1.2 ± 0.5	1.2 ± 0.3	1.3 ± 0.5	2.5 ± 1.3	1.6 ± 0.6	1.8 ± 0.7	2.1 ± 1.1
Basophils (%)	0.3 ± 0.1	0.3 ± 0.1	0.3 ± 0.1	0.2 ± 0.1	0.2 ± 0.1	0.1 ± 0.1	0.1 ± 0.1	0.1 ± 0.1
LUC (%)	1.2 ± 0.4	1.4 ± 0.3	1.2 ± 0.3	1.1 ± 0.5	1.1 ± 0.5	1.2 ± 0.3	1.1 ± 0.2	1.2 ± 0.2
No. of rats examined	10	10	10	10	10	10	10	10

^1^ Only parameters with statistical significance or tendency are shown in table. ^2^ Data are expressed as mean ± SD. * *p* < 0.05 and ** *p* < 0.01 compared with the control. Bold values indicate statistical significance. LUC, Large unstained cells; WBC, White blood cell count.

**Table 4:** Summary of selected ^1^ serum biochemistry findings in the 26-week repeated-dose oral toxicity study of *Scutellaria baicalensis* extract.

Parameter (Units)	Groups (mg/kg/Day)							
	Male				Female			
	0	500	1000	2000	0	500	1000	2000
Triglyceride (mg/dL)	73.6 ± 40.9 ^2^	89.8 ± 20.0	71.2 ± 20.2	88.4 ± 15.6	38.4 ± 6.3	42.1 ± 10.6	40.2 ± 7.7	49.4 ± 8.2 *
Albumin (g/dL)	3.17 ± 0.17	3.10 ± 0.10	3.06 ± 0.09	3.08 ± 0.12	3.40 ± 0.14	3.33 ± 0.19	3.27 ± 0.25	3.29 ± 0.16
Creatinine (mg/dL)	0.52 ± 0.03	0.54 ± 0.03	0.50 ± 0.03	0.51 ± 0.03	0.61 ± 0.05	0.58 ± 0.04	0.59 ± 0.08	0.57 ± 0.07
Ca^2+^ (mg/dL)	10.17 ± 0.27	10.15 ± 0.28	9.94 ± 0.27	9.91 ± 0.38	9.91 ± 0.26	9.56 ± 0.50	9.69 ± 0.35	9.58 ± 0.29
No. of rats examined	10	10	10	10	10	10	10	10

^1^ Only parameters with statistical significance or tendency are shown in table. ^2^ Data are expressed as mean ± SD. * *p* < 0.05 compared with the control. Bold values indicate statistical significance.

**Table 5:** Plasma coagulation values of male and female rats in the 26-week repeated-dose oral toxicity study of *Scutellaria baicalensis* extract.

Parameter (Units)	Groups (mg/kg/Day)							
	Male				Female			
	0	500	1000	2000	0	500	1000	2000
PT (s)	16.04 ± 0.73 ^1^	15.74 ± 0.57	16.30 ± 1.15	15.62 ± 1.10	15.94 ± 0.58	16.23 ± 0.38	16.48 ± 0.46	16.22 ± 0.45
aPTT (s)	18.46 ± 1.10	18.55 ± 1.03	18.12 ± 1.41	17.65 ± 1.31	17.72 ± 2.05	18.48 ± 1.37	18.64 ± 0.68	19.11 ± 1.05
No. of rats examined	10	10	10	10	10	10	10	10

^1^ Data are expressed as mean ± SD. aPTT, activated partial thromboplastin time; PT, Prothrombin time.

**Table 6:** Summary of selected ^1^ absolute and relative organ weights in the 26-week repeated-dose oral toxicity study of *Scutellaria baicalensis* extract.

Organ (Units)	Groups (mg/kg/Day)							
	Male				Female			
	0	500	1000	2000	0	500	1000	2000
Liver (g)	12.4515 ± 0.8402 ^2^	13.6710 ± 1.6110	12.8915 ± 1.4997	14.2316 ± 1.9118	6.0878 ± 0.5471	6.3077 ± 0.6146	6.3630 ± 0.5781	6.8665 ± 0.6300 **
Per body weight (%)	2.3356 ± 0.2268	2.5531 ± 0.1444 *	2.5494 ± 0.1841 *	2.6852 ± 0.2140 *	2.2780 ± 0.1886	2.3489 ± 0.2077	2.3609 ± 0.2004	2.5627 ± 0.2742 *
Spleen (g)	0.8666 ± 0.0771	0.9255 ± 0.1230	0.8723 ± 0.1452	0.9444 ± 0.3031	0.5673 ± 0.0427	0.5430 ± 0.0555	0.5900 ± 0.0759	0.5772 ± 0.0653
Per body weight (%)	0.1630 ± 0.0219	0.1740 ± 0.0244	0.1728 ± 0.0275	0.1776 ± 0.0479	0.2121 ± 0.0119	0.2021 ± 0.0170	0.2187 ± 0.0249	0.2146 ± 0.0181
Kidney-left (g)	1.5500 ± 0.1068	1.6006 ± 0.1460	1.5420 ± 0.1653	1.6989 ± 0.2075	0.7883 ± 0.0488	0.7801 ± 0.0403	0.7856 ± 0.0593	0.7914 ± 0.0447
Per body weight (%)	0.2909 ± 0.0302	0.2998 ± 0.0197	0.3053 ± 0.0229	0.3211 ± 0.0277	0.2959 ± 0.0296	0.2913 ± 0.0242	0.2919 ± 0.0262	0.2953 ± 0.0223
Kidney-right (g)	1.5568 ± 0.1044	1.5971 ± 0.1531	1.5148 ± 0.1814	1.5921 ± 0.3106	0.7820 ± 0.0409	0.7824 ± 0.0519	0.7907 ± 0.0370	0.7893 ± 0.0514
Per body weight (%)	0.2922 ± 0.0305	0.2989 ± 0.0169	0.2998 ± 0.0259	0.3031 ± 0.0611	0.2931 ± 0.0215	0.2918 ± 0.0229	0.2938 ± 0.0189	0.2947 ± 0.0268
Adrenal gland-left (g)	0.0263 ± 0.0040	0.0308 ± 0.0031	0.0286 ± 0.0044	0.0266 ± 0.0041	0.0642 ± 0.1016	0.0319 ± 0.0027	0.0328 ± 0.0044	0.0300 ± 0.0046
Per body weight (%)	0.0049 ± 0.0007	0.0058 ± 0.0008 *	0.0057 ± 0.0008	0.0050 ± 0.0008	0.0247 ± 0.0401	0.0119 ± 0.0009	0.0122 ± 0.0017	0.0112 ± 0.0019
Adrenal gland-right (g)	0.0256 ± 0.0044	0.0294 ± 0.0044	0.0274 ± 0.0032	0.0261 ±0.0030	0.0293 ± 0.0047	0.0288 ± 0.0029	0.0292 ± 0.0026	0.0278 ± 0.0043
Per body weight (%)	0.0048 ± 0.0010	0.0055± 0.0009	0.0054 ± 0.0006	0.0049 ± 0.0006	0.0110 ± 0.0021	0.0107 ± 0.0012	0.0109 ± 0.0011	0.0104 ± 0.0018
No. of rats examined	10	10	10	10	10	10	10	10

^1^ Only parameters with statistical significance or tendency are shown in table. ^2^ Data are expressed as mean ± SD. * *p* < 0.05 and ** *p* < 0.01 compared with the control. Bold values indicate statistical significance.

## Data Availability

The datasets generated and analyzed during the current study are available from the corresponding author upon reasonable request.

## Supplementary Materials

The following supporting information can be downloaded at https://www.mdpi.com/article/10.3390/toxics14100919/s1, Table S1: Histopathological findings in male and female rats from the main groups of the 26-week repeated-dose oral toxicity study of *Scutellaria baicalensis* extract; Table S2: Histopathological findings in male and female rats from the recovery groups of the 26-week repeated-dose oral toxicity study of *Scutellaria baicalensis* extract; Table S3: Body and liver weights of male rats in the main groups of the 26-week repeated-dose oral toxicity study of *Scutellaria baicalensis* extract.

## Abbreviations

The following abbreviations are used in this manuscript:

aPTT	Activated partial thromboplastin time
CBC	Complete blood count
EDTA	Ethylenediaminetetraacetic acid
FDA	U.S. Food and Drug Administration
GLP	Good Laboratory Practice
ICH	International Council for Harmonisation
LUC	Large unstained cells
MLD	Minimum lethal dose
NOAEL	No-observed-adverse-effect level
NOEL	No-observed-effect level
PT	Prothrombin time
SBE	*Scutellaria baicalensis* extract
SD	Sprague–Dawley
SD	Standard deviation
SPSS	Statistical Package for the Social Sciences
WBC	White blood cell
