# Supplementary material for: Safety Evaluation of Scutellaria baicalensis: Acute and 26-Week Repeated-Dose Oral Toxicity Studies in Rats
Source: Toxics. 2026 Oct 17;14(10):919. doi: 10.3390/toxics14100919 (PMC13709419; doi:10.3390/toxics14100919)
Supplement: Supplementary file 1 [file toxics-14-00919-s001.zip › toxics-4571147-supplementary.pdf]

## Supplementary material

**Table S1.** Histopathological findings in male and female rats from the main groups of the 26-week repeated-dose oral toxicity study of *Scutellaria baicalensis* extract.

| Organs | Findings | Groups (mg/kg/day) | | | |
|---|---|---|---|---|---|
| | | 0 | 500 | 1000 | 2000 |
| **Male** | | | | | |
| No. of male rats examined | | 10 | 0 | 1 | 10 |
| Adrenal gland | Lipidosis, cortical | 3 (30) [1] [2/1/0/0] [2] | NE | NE | 1 (10) [1/0/0/0] |
| Kidney | Hyaline cast | 8 (80) [6/1/1/0] | NE | NE | 7 (70) [4/3/0/0] |
| | Chronic progressive nephrosis | 3 (30) [2/1/0/0] | NE | NE | 0 (0) |
| Lung | Alveolar histiocytosis, multifocal | 1 (10) [1/0/0/0] | NE | NE | 0 (0) |
| | Perivascular lymphoid cell infiltration | 0 (0) | NE | NE | 1 (10) [1/0/0/0] |
| Heart | Myocarditis, focal | 0 (0) | NE | NE | 1 (10) [1/0/0/0] |
| Thymus | Hemorrhage | 3 (30) [1/2/0/0] | NE | 1 (100) [1/0/0/0] | 1 (10) [1/0/0/0] |
| Thyroid gland | Ultimobranchial cyst | 4 (40) [1/3/0/0] | NE | NE | 1 (10) [0/0/1/0] |
| | Hemorrhage | 0 (0) | NE | NE | 1 (10) [1/0/0/0] |
| Mandibular lymph node | Sinus erythrocytosis | 0 (0) | NE | NE | 3 (30) [2/1/0/0] |
| Mesenteric lymph nodes | Sinus erythrocytosis | 4 (40) [3/1/0/0] | NE | NE | 2 (20) [2/0/0/0] |
| Stomach | Squamous cell hyperplasia, focal | 1 (10) [1/0/0/0] | NE | NE | 2 (20) [0/2/0/0] |
| Brain | Cyst formation | 0 (0) | NE | NE | 1 (10) [0/1/0/0] |
| **Female** | | | | | |
| No. of female rats examined | | 10 | 1 | 2 | 10 |
| Pituitary gland | Cyst-like structure, pars intermedia | 1 (10) [1/0/0/0] | NE | NE | 0 (0) |
| Ovary | Follicular cyst | 1 (10) [0/1/0/0] | NE | NE | 3 (30) [1/2/0/0] |
| | Cystic corpus luteum | 1 (10) [0/1/0/0] | NE | NE | 0 (0) |
| | Luteal cyst | 0 (0) | NE | NE | 2 (20) [0/0/2/0] |
| Uterus | Squamous metaplasia | 2 (20) [2/0/0/0] | 0 (0) | NE | 3 (30) [3/0/0/0] |
| | Epidermoid cyst | 0 (0) | 1 (100) [0/0/1/0] | NE | 0 (0) |
| Kidney | Hyaline cast | 5 (50) | NE | NE | 4 (40) |

| | | | | | |
|---|---|---|---|---|---|
| | | [2/2/1/0] | | | [1/3/0/0] |
| | Chronic progressive nephrosis | 0 (0) | NE | NE | 3 (30) [3/0/0/0] |
| Lung | Alveolar histiocytosis, multifocal | 1 (10) [0/1/0/0] | NE | NE | 1 (10) [0/1/0/0] |
| Thymus | Hemorrhage | 1 (10) [1/0/0/0] | NE | 1 (100) [1/0/0/0] | 1 (10) [1/0/0/0] |
| Thyroid gland | Ultimobranchial cyst | 2 (20) [0/2/0/0] | NE | NE | 1 (10) [1/0/0/0] |
| Mandibular lymph node | Sinus erythrocytosis | 0 (0) | NE | 1 (100) [0/0/1/0] | 0 (0) |

[1] Values indicate the number of rats with each finding, with percentages in parentheses calculated using the number of rats in which the respective organ was examined.

[2] Bracketed values indicate the numbers of rats with findings graded 1+ (minimal), 2+ (mild), 3+ (moderate), and 4+ (marked), respectively.

NE, not examined. At 500 and 1000 mg/kg/day, only organs with gross abnormalities were examined.

**Table S2.** Histopathological findings in male and female rats from the recovery groups of the 26-week repeated-dose oral toxicity study of *Scutellaria baicalensis* extract.

| **Organs** | **Findings** | **Groups (mg/kg/day)** | |
|---|---|---|---|
| | | **0** | **2000** |
| **Male** | | | |
| No. of male rats examined | | 5 | 5 |
| Adrenal gland | Accessory adrenocortical tissue | 1 (20) [1] [1/0/0/0] [2] | 0 (0) |
| Kidney | Hyaline cast | 4 (80) [1/3/0/0] | 5 (100) [2/1/2/0] |
| | Chronic progressive nephropathy | 1 (20) [1/0/0/0] | 1 (20) [0/1/0/0] |
| Pancreas | Lobular atrophy | 0 (0) | 1 (20) [0/1/0/0] |
| Lung | Alveolar histiocytosis, multifocal | 3 (60) [3/0/0/0] | 0 (0) |
| Thymus | Hemorrhage | 0 (0) | 1 (20) [1/0/0/0] |
| Thyroid gland | Ultimobranchial cyst | 1 (20) [0/1/0/0] | 1 (20) [0/1/0/0] |
| Pituitary gland | Cyst-like structure, pars intermedia | 0 (0) | 1 (20) [0/1/0/0] |
| **Female** | | | |
| No. of female rats examined | | 5 | 5 |
| Ovary | Follicular cyst | 2 (40) [0/2/0/0] | 1 (20) [0/1/0/0] |
| | Luteal cyst | 1 (20) [0/1/0/0] | 1 (20) [0/1/0/0] |
| | Cystic corpus luteum | 1 (20) [0/1/0/0] | 0 (0) |
| Uterus | Epidermoid cyst | 1 (20) [0/1/0/0] | 0 (0) |
| | Glandular cyst | 0 (0) | 1 (20) [0/1/0/0] |

| | | | |
|---|---|---|---|
| Kidney | Hyaline cast | 2 (40) [1/1/0/0] | 0 (0) |
| | Pyelonephritis | 1 (20) [0/1/0/0] | 0 (0) |
| Urinary bladder | Cystitis | 1 (20) [0/1/0/0] | 0 (0) |
| Liver | Inflammatory cell foci, multifocal | 1 (20) [1/0/0/0] | 0 (0) |
| Lung | Alveolar histiocytosis, multifocal | 1 (20) [0/1/0/0] | 0 (0) |
| Thymus | Hemorrhage | 2 (40) [2/0/0/0] | 0 (0) |
| Mandibular lymph node | Sinus erythrocytosis | 1 (20) [0/1/0/0] | 0 (0) |
| Brain | Cyst formation | 1 (20) [0/1/0/0] | 0 (0) |

[1] Values indicate the number of rats with each finding, with percentages in parentheses calculated using the number of rats in which the respective organ was examined.

[2] Bracketed values indicate the numbers of rats with findings graded 1+ (minimal), 2+ (mild), 3+ (moderate), and 4+ (marked), respectively.

**Table S3.** Body and liver weights of male rats in the main groups of the 26-week repeated-dose oral toxicity study of *Scutellaria baicalensis* extract.

| **Parameter (unit)** | **Groups (mg/kg/day)** | | | |
|---|---|---|---|---|
| | **0** | **500** | **1000** | **2000** |
| Body weight at day 0 (g) | 188.09 ± 4.61 | 187.94 ± 6.96 (−0.08%) | 188.02 ± 5.53 (−0.04%) | 188.07 ± 4.55 (−0.01%) |
| Body weight at week 26 (g) | 547.60 ± 34.74 | 554.08 ± 49.94 (+1.18%) | 523.21 ± 29.15 (−4.45%) | 547.96 ± 38.78 (+0.07%) |
| Body weight gain, weeks 0–26 (g) [1] | 359.51 ± 34.12 | 366.15 ± 44.73 (+1.85%) | 335.19 ± 28.91 (−6.77%) | 359.89 ± 35.30 (+0.11%) |
| Terminal body weight (g) [2] | 535.71 ± 42.00 | 534.67 ± 46.68 (−0.20%) | 504.54 ± 29.08 (−5.82%) | 528.54 ± 36.51 (−1.34%) |
| Absolute liver weight (g) | 12.4515 ± 0.8402 | 13.6710 ± 1.6110 (+9.79%) | 12.8915 ± 1.4997 (+3.53%) | 14.2316 ± 1.9118 (+14.30%) |
| Relative liver weight (%) [3] | 2.3356 ± 0.2268 | **2.5531 ± 0.1444* (+9.31%)** | **2.5494 ± 0.1841* (+9.16%)** | **2.6852 ± 0.2140* (+14.97%)** |
| No. of rats examined [4] | 10 | 10 | 10 | 10 |

[1] Body weight gain was calculated for each animal as body weight at week 26 minus body weight at day 0.

[2] Terminal body weight was measured immediately before necropsy after overnight fasting and is distinct from the week 26 body weight.

[3] Relative liver weight was calculated for each animal as (liver weight/terminal body weight) × 100.

[4] Only males in the main groups were included; recovery animals were excluded.

Data are expressed as mean ± SD ($n$ = 10 per group).

Values in parentheses indicate the percentage difference from the control mean, calculated as [(treated mean/control mean) − 1] × 100.

* $p < 0.05$ versus the control. Bold values indicate statistical significance.
